# AML Metabolism: A Dynamic Continuum of Functional Dependencies

**DOI:** 10.3390/cells15211955

**Published:** 2026-10-27

**Authors:** Giuseppe Zardo, Gloria Pasqualini, Gianfranco Catalano, Alessandra Zaza, Nelida Ines Noguera

**Affiliations:** 1 Interdisciplinary Department of Wellbeing, Health and Environmental Sustainability (BeSSA), Sapienza University of Rome, Complesso Santa Lucia—Via Delle Fontanelle snc, 02100 Rieti, Italy; giuseppe.zardo@uniroma1.it; 2 LIFE Institute for Research and Health Care—Santa Lucia IRCCS, Via del Fosso di Fiorano 64, 00143 Rome, Italy; glorishina22@gmail.com (G.P.); gianfranco.catalano@uniroma2.it (G.C.); zazaalessandra96@gmail.com (A.Z.); 3 Department of Biomedicine and Prevention, University of Rome Tor Vergata, Via Montpellier 1, 00133 Rome, Italy

**Keywords:** acute myeloid leukemia, glycolysis, redox, metabolic dependencies, leukemic stem cells, oxidative phosphorylation, acute promyelocytic leukemia, metabolic heterogeneity, fatty acid oxidation, therapeutic resistance

## Abstract

**Highlights:**

**What are the main findings?**

- AML cells occupy a dynamic continuum of metabolic states, with distinct and context-dependent dependencies in HSCs, LSCs, and leukemic blasts.
- LSC OXPHOS dependence differs from adaptive mitochondrial rewiring in therapy-resistant AML, while PML::RARA-driven APL exhibits a metabolically constrained state characterized by enhanced FAO, OXPHOS, and arginine-associated metabolism.

**What are the implications of the main findings?**

- Metabolic activity alone is insufficient to define AML subgroups; functional dependencies and potential metabolic escape routes should guide the identification of metabolic biomarkers.
- Integrating metabolic profiling with genomic, transcriptomic, proteomic, and metabolomic data may enable biomarker-guided combination therapies targeting metabolic vulnerabilities and therapeutic resistance.

**Abstract:**

Metabolic reprogramming is increasingly recognized as a fundamental component of acute myeloid leukemia (AML) biology, but AML cannot be defined by a single metabolic phenotype. Leukemic cells occupy dynamic metabolic states shaped by differentiation, genetic background, the bone marrow microenvironment, and therapeutic pressure. In this Review, we propose that metabolic biomarkers should capture functional dependencies and metabolic plasticity rather than static metabolic states, with functional dependencies defined as the reliance of AML cells on a specific metabolic pathway or process for their survival, proliferation or maintenance, rather than a simple glycolytic-versus-oxidative dichotomy. Normal hematopoietic stem cells (HSCs), leukemic stem cells (LSCs), and bulk AML blasts display distinct metabolic programs. Quiescent HSCs maintain a low-energy state, whereas LSCs rely strongly on mitochondrial oxidative phosphorylation (OXPHOS), amino acid metabolism, mitochondrial quality control, and antioxidant defenses. AML blasts exhibit greater metabolic heterogeneity and can switch between glycolytic and oxidative pathways. Importantly, increased mitochondrial activity does not necessarily indicate an LSC phenotype, as therapy-resistant AML cells may acquire enhanced OXPHOS, fatty acid oxidation (FAO), and mitochondrial fitness as adaptive states. Acute promyelocytic leukemia (APL) provides a model of lineage-specific metabolic rewiring, with PML::RARA suppressing glycolysis while promoting FAO, TCA cycle activity, OXPHOS, and arginine-associated anaplerosis. We propose that metabolic biomarkers should identify functional dependencies and escape routes. Integrating metabolic profiling with multi-omics approaches may enable identification of AML subgroups with distinct vulnerabilities and guide rational combination therapies.

## 1. Hematopoiesis and Metabolic States in Normal and Leukemic Cells

### 1.1. Hematopoiesis and Hematopoietic Metabolism

Over the past decade, increasing attention has been directed toward the selective targeting of cancer cell metabolism. This interest stems from the recognition that metabolic reprogramming constitutes a fundamental hallmark of malignant transformation [1]. Metabolic remodeling sustains tumor initiation and progression by enhancing the acquisition, synthesis, and utilization of key nutrients. In AML, these changes involve dysregulation of glycolytic flux, mitochondrial oxidative phosphorylation (OXPHOS), lipidic and AA metabolism, collectively enabling leukemic cells to proliferate and survive under diverse environmental and therapeutic stresses [2,3].

Traditionally, hematopoiesis has been described as a strictly hierarchical process in which hematopoietic stem cells (HSCs) give rise to progressively more lineage-restricted progenitors, ultimately generating differentiated blood cells [4,5]. However, recent single-cell studies have challenged this model, demonstrating that HSCs and their downstream progeny do not exist as discrete, well-defined cell types. Instead, they occupy a continuum of transitional states, with gradual shifts in gene expression and lineage potential. These findings support a paradigm in which hematopoiesis is a continuous differentiation process rather than a rigid, stepwise hierarchy [6,7,8]. HSCs are heterogeneous and exhibit variability in both self-renewal capacity and lineage-specific differentiation potential [9,10,11]. This heterogeneity arises from both intrinsic cellular and epigenetic variation and from extrinsic cues, including signals from the bone marrow microenvironment, niche-derived cytokines, and systemic stimuli such as stress or inflammation [12,13,14,15,16,17,18,19]. Thus, intrinsic and extrinsic factors generate functional diversity among HSCs, shaping their self-renewal, lineage bias, and contribution to physiological hematopoiesis, while variability may promote leukemogenic drift and influence therapeutic outcomes. HSCs also exhibit pronounced metabolic heterogeneity, which influences their self-renewal, lineage commitment, and leukemic outcome [20,21,22].

Recent evidence indicates that metabolic reprogramming is not only essential for normal hematopoietic stem cell fate decisions, but also underlies leukemogenesis. For example, metabolic rewiring of energy pathways and mitochondrial function differs between normal HSCs and leukemic stem cells (LSCs), influencing leukemic development and progression [23,24,25]. Chromosomal rearrangements, as well as genetic mutations and epigenetic alterations arising in hematopoietic stem and/or progenitor cells, drive leukemic transformation and give rise to cells known as LSCs or leukemia-initiating cells (LICs), which sustain chronic myeloid leukemia (CML) and AML [26].

### 1.2. Definitions and Isolation of HSC, LSC and Bulk AML Populations

Direct comparison of metabolite profiles across HSCs, LSCs and bulk AML cells is complicated by the lack of a universally accepted definition of the leukemic stem-cell compartment. LSC-enriched populations have been isolated by immunophenotypic criteria—most commonly CD34^+^CD38^−^ cells, further refined with CD123, TIM-3, CLEC12A, CD96 or CD44—or by functional enrichment of ROS-low cells. ROS-low sorting has been used in metabolomic studies to obtain LSC-enriched fractions, often showing concordance with CD34^+^CD38^−^CD123^+^ phenotypic LSCs, but it simultaneously selects for a specific redox state and may influence measured metabolite abundance. Conversely, “bulk” AML populations have been variably defined as total blasts, CD34^+^ fractions or ROS-high cells [23,24]. These differences in cell selection, together with patient genotype, treatment exposure and metabolomic methodology, should be considered when integrating metabolite data across studies.

### 1.3. HSCs, LSCs and Leukemic Blasts Exhibit Distinct Metabolic Programs

HSCs operate under reduced energetic demand, exhibiting lower oxygen consumption, diminished ATP production, and a metabolic profile biased toward glycolysis rather than oxidative phosphorylation, preserving quiescence and stem cell function [27,28,29]. AML blasts exhibit increased metabolic activity via enhanced glycolytic flux and PPP (pentose phosphate pathway) engagement [30], with upregulated key enzymes (e.g., HK2, G6PD) in AML cell lines [31], and a role in supporting leukemic cell growth and survival, since inhibiting PPP activity reduces proliferation and migration [32]. This supports increased ATP production and provision of biosynthetic precursors (e.g., ribose phosphates, NADPH) necessary for the unchecked proliferation and maintenance of leukemic blasts [33,34].

**Figure 1:**
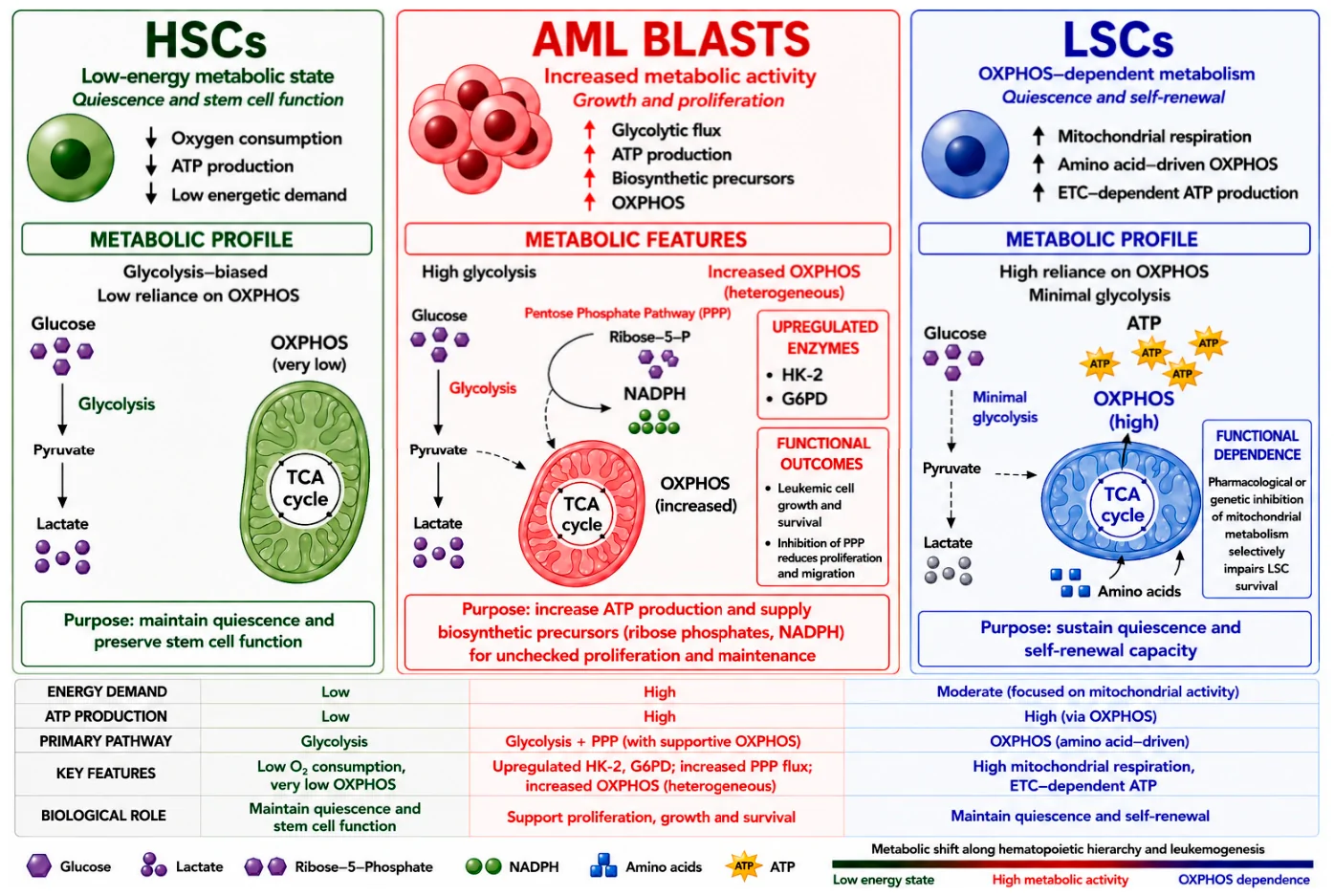
Metabolic features of HSCs, AML blasts and LSCs. HSCs exhibit a low-energy metabolic state supporting quiescence, whereas AML blasts display increased glycolytic, PPP and heterogeneous OXPHOS activity to sustain proliferation and survival. LSCs are characterized by strong mitochondrial OXPHOS dependence, supporting quiescence and self-renewal.

On the contrary LSCs exhibit a pronounced reliance on OXPHOS to sustain their quiescence and self-renewal capacity. Primary AML specimens and cell line models demonstrate that LSCs display elevated mitochondrial respiration, AA–driven OXPHOS, and electron transport chain–dependent ATP production. Pharmacological or genetic perturbation of mitochondrial metabolism selectively impairs LSC survival, highlighting the functional necessity of mitochondrial pathways in these cells [21,35,36] (Figure 1).

It is important to note that metabolic features such as increased glycolysis and glutaminolysis are not exclusive to AML cells. Resting and activated immune cells, particularly T lymphocytes, exhibit distinct metabolic programs according to their activation and differentiation states. Therefore, shared metabolic features should be interpreted in the context of cellular identity and biological function, rather than considered disease-specific characteristics [37].

The metabolic landscape of AML is shaped by the interplay between genetic background, cellular differentiation state, and functional identity. Although normal HSCs, LSCs, and bulk AML blasts exhibit distinct metabolic characteristics, these programs are not fixed and may overlap or change in response to microenvironmental signals and therapeutic pressure [23,24,25]. In particular, LSC-enriched populations can display a strong dependence on amino-acid-supported oxidative phosphorylation, whereas chemotherapy-resistant AML cells may acquire increased oxidative metabolism and fatty acid oxidation without necessarily exhibiting functional LSC properties [38,39,40]. Genetic alterations can further influence metabolic dependencies, as illustrated by genotype-associated metabolic heterogeneity in AML [2] and by the distinct metabolic rewiring observed in PML::RARA-positive acute promyelocytic leukemia [41]. Table 1 integrates representative genetic backgrounds, differentiation and LSC states, and their reported metabolic dependencies, highlighting how these factors contribute to the dynamic metabolic continuum of AML.

## 2. Glucose Metabolism and Metabolic Signaling in AML

### 2.1. Glycolytic Metabolism and the Pentose Phosphate Pathway

The Warburg effect, commonly observed in cancer cells, is characterized by preferential conversion of glucose-derived pyruvate to lactate despite sufficient oxygen availability. Instead of undergoing mitochondrial oxidative decarboxylation to acetyl-CoA and subsequent oxidation in the tricarboxylic acid (TCA) cycle, pyruvate is largely reduced to lactate. The elevated glycolytic throughput facilitates NAD^+^ regeneration and supplies intermediates for neoplastic growth [49]. Glucose metabolism is interconnected with several ancillary pathways, including the PPP, which provides ribose-5-phosphate for nucleotide biosynthesis and generates NADPH; the serine biosynthesis pathway, which contributes AAs and one-carbon units; and folate-mediated one-carbon metabolism, which supports nucleotide synthesis and redox homeostasis through NADPH production. Consequently, glycolytic flux not only sustains ATP generation but also supplies essential metabolic intermediates that function as precursors for macromolecular biosynthesis in proliferating cancer cells [49,50,51].

### 2.2. GLUT Family Expression in HSCs, LSCs and AML Blasts

All hematopoietic cells rely on transporters of the GLUT family to import glucose, but the isoforms expressed vary according to cell type. Quiescent HSCs express GLUT1 at basal levels to support low-level glycolysis and oxidative phosphorylation, whereas progenitor cells upregulate GLUT1 to sustain increased glycolytic flux during proliferation [52,53]. Although GLUT3 has been described in other stem cell types (e.g., neural stem cells) and in activated immune cells, direct evidence of its expression in normal HSCs or progenitors in the bone marrow remains unknown [54,55]. AML blasts and LSCs overexpress GLUT1, which drives elevated glycolytic flux, lactate production, and chemoresistance [52,56]. Consequently, high levels of pyruvate kinase M2 (PKM2) and lactate dehydrogenase A (LDHA) are detected in AML blasts and LSCs, where they support glycolytic metabolism, NAD^+^ regeneration, and cell survival [23,42]. To note that elevated PKM2 and LDHA activity, together with increased pyruvate and lactate levels, have been associated with poor prognosis in AML patients [24]. Deletion or inhibition of PKM2 selectively impairs progenitor and LSC proliferation without affecting quiescent HSCs, whereas LDHA loss disrupts glycolysis in both progenitors and HSCs. In addition, glycolysis is controlled through a network of signaling molecules including LKB1-AMPK and PI3K/AKT/mTOR [24,57,58].

### 2.3. LKB1-AMPK Signaling

AMP-activated protein kinase (AMPK), a central regulator of cellular energy homeostasis, is activated under conditions of energetic stress, leading the Liver kinase B1 (LKB1, STK11) to phosphorylate Thr172 within the AMPK1 catalytic α-subunit. A major downstream effect is inhibition of mechanistic target of rapamycin complex 1 (mTORC1) via phosphorylation of TSC2 and Raptor, limiting protein synthesis and cell growth under low-energy conditions [59,60]. Deletion of LKB1 results in rapid exhaustion of the HSC murine pool, loss of quiescence, and impaired hematopoiesis [51,52]. In contrast, deletion of AMPK induces reduced ATP levels and mitochondrial alterations, but does not lead to rapid HSC exhaustion or loss of regenerative capacity, indicating a milder phenotype [61]. Nonetheless, AMPK contributes to mitochondrial integrity and metabolic adaptation under stress conditions [62].

**Figure 2:**
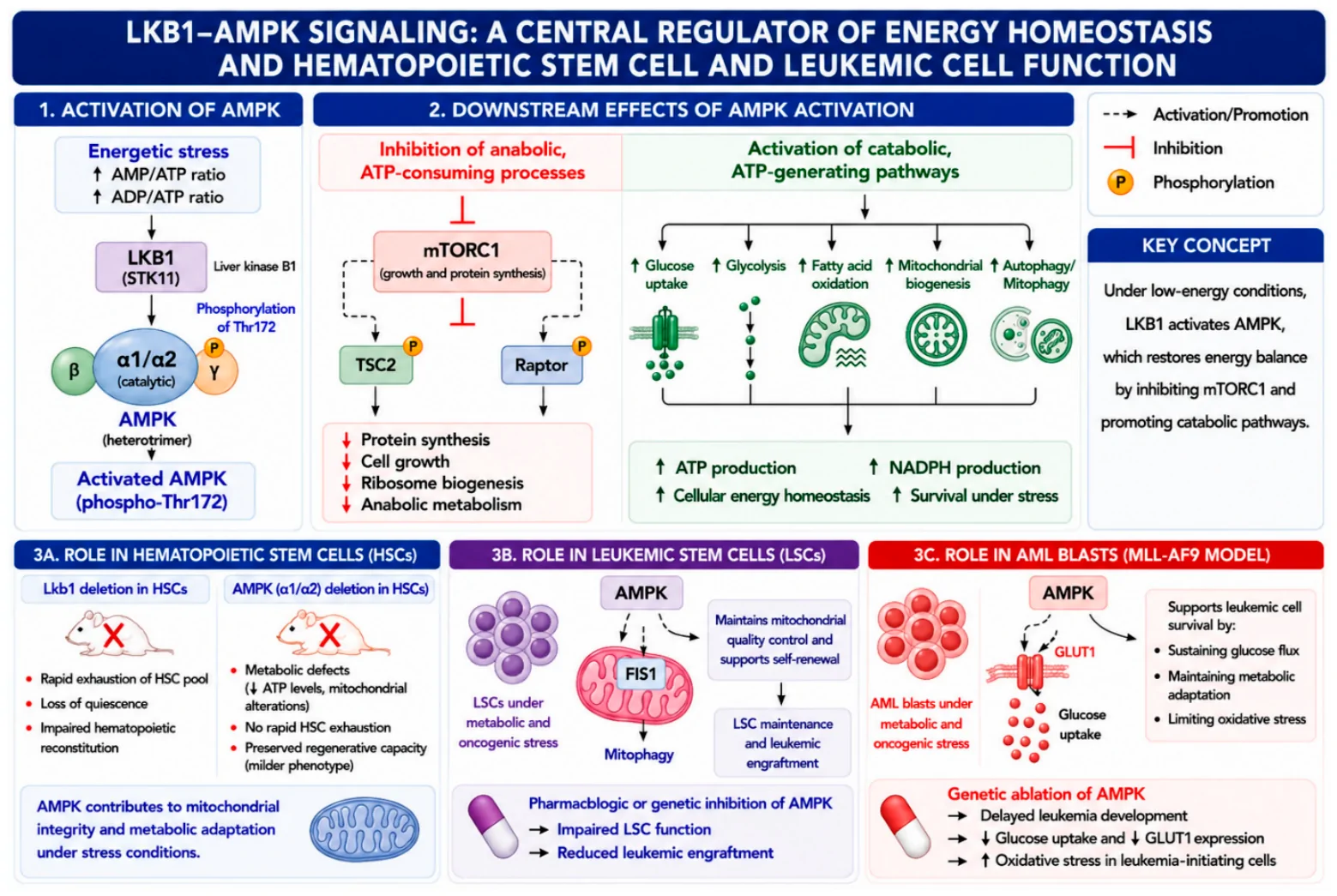
LKB1–AMPK signaling in energy homeostasis and hematopoietic and leukemic stem cell function. Under energetic stress, LKB1 activates AMPK, which inhibits ATP-consuming anabolic processes through mTORC1 suppression while promoting catabolic pathways to restore cellular energy balance. AMPK supports mitochondrial integrity and adaptation in HSCs and contributes to LSC maintenance and self-renewal, whereas in AML blasts it sustains metabolic fitness and survival.

LSCs and AML blasts operate under increased metabolic stress. In a murine MLL-AF9 AML model, genetic ablation of AMPK delayed leukemia development, reduced glucose uptake, decreased GLUT1 expression, and increased oxidative stress in leukemia-initiating cells. Thus, AMPK supports leukemic cell survival by sustaining glucose flux and metabolic adaptation under stress conditions. Pei et al. showed that AMPK activity is required for the self-renewal of human LSCs through regulation of FIS1-mediated mitophagy [63]. Pharmacologic or genetic inhibition of AMPK impairs LSC function and leukemic engraftment capacity, demonstrating that AMPK sustains mitochondrial quality control programs essential for LSC maintenance [63] (Figure 2).

### 2.4. PI3K/AKT/mTOR Signaling

Persistent AKT activation in murine hematopoietic progenitors induces myeloproliferative disease and leukemic transformation while depleting the normal HSC pool [64]. Xu et al. demonstrated that survival of primary AML blasts depends on PI3K activation, and pharmacologic inhibition significantly reduced blast viability [65].

In murine models, Yilmaz et al. demonstrated that deletion of PTEN induced leukemogenesis through mTOR activation, and that rapamycin treatment eliminated leukemia-initiating cells while restoring normal hematopoiesis. These findings indicate that, in the leukemic context, mTORC1 activity is essential for LSC maintenance, whereas mTORC1 activity is more tightly restrained in normal HSCs under homeostatic conditions [66]. We demonstrated that in AML, mutant NPM1 (NPM1c+) disrupts the interaction between PTEN and the deubiquitinase HAUSP (USP7), promoting PTEN cytoplasmic sequestration, polyubiquitination, and degradation, thereby impairing its tumor suppressor function and favoring PI3K/AKT pathway activation [44]. In acute promyelocytic leukemia (APL), we identified PTEN as a direct transcriptional target repressed by the PML::RARα fusion protein through inhibition of PU.1 activity. Treatment with all-trans retinoic acid (ATRA) restored PTEN expression and nuclear localization by inducing an active chromatin state at the PTEN promoter, leading to modulation of downstream effectors, including Aurora kinase A [67]. Together, these studies established novel transcriptional and post-translational mechanisms controlling PTEN expression, stability, and subcellular localization in AML.

Tight regulation of PI3K/AKT/mTOR signalling is required for normal HSC maintenance, whereas persistent activation confers a selective survival and self-renewal advantage to LSCs.

### 2.5. Crosstalk Between LKB1-AMPK and PI3K/AKT/mTOR Signaling Pathways

In HSCs, energetic stress characterized by an increased AMP/ATP ratio activates the LKB1-AMPK axis; thus, AMPK, in turn, phosphorylates Raptor and TSC2 (critical components of the mTORC1 and TSC2 complexes, respectively), leading to inhibition of mTORC1. This reduces protein synthesis, promotes autophagy, and preserves metabolic homeostasis, maintaining quiescence and long-term stem cell function [57,58]. In LSCs and AML blasts, AMPK can also be activated in responses to metabolic stress and supports survival through context-dependent mechanisms. Although AMPK can inhibit mTORC1 through phosphorylation of Raptor and TSC2, the extent and functional consequences of this inhibition depend on the cellular and metabolic context. In leukemic cells, additional oncogenic signaling, including PI3K/AKT activation, can sustain mTORC1 activity despite AMPK activation. At the same time, AMPK promotes OXPHOS, PPP, mitophagy, and maintenance of ATP levels, enabling metabolic adaptation and resistance to chemotherapeutic stress. Thus, AMPK signaling has distinct functional outcomes depending on cellular context, preserving quiescence in normal HSCs while supporting survival and metabolic flexibility in LSCs and AML blasts [60,63].

## 3. Mitochondrial Metabolism in HSCs, LSCs and AML Blasts

### 3.1. TCA Cycle and Mitochondrial Substrate Utilization

TCA cycle transfers high-energy electrons via NADH and FADH to the electron transport chain (ETC), linking glycolysis to mitochondrial ATP production [68]. The TCA cycle is initiated by the end-product of glycolysis, pyruvate, which is converted into acetyl-CoA. In AML cells, since glycolysis and the TCA cycle are uncoupled, pyruvate is converted into lactate; the TCA cycle utilizes alternative substrates (Warburg effect). Activation of PI3K/AKT/mTOR and stabilization of hypoxia-inducible factor 1α (HIF-1α) enhance the transcription of genes encoding GLUT1, HK2, and LDHA, thereby promoting glycolysis while simultaneously repressing OXPHOS. LDHA, converting pyruvate to lactate, is crucial for maintaining glycolytic flux in AML cells and regenerating cytosolic NAD^+^ required for sustained glycolysis. LDHA inhibition in AML cell lines and primary patient samples leads to decreased glycolytic activity, disrupted NAD^+^/NADH balance, and cell death, highlighting a reliance on aerobic glycolysis and diversion of pyruvate away from mitochondrial oxidation [69]. AML models have shown that the activity of pyruvate dehydrogenase kinases (PDKs) prevents pyruvate entry into mitochondria, thereby sustaining glycolytic activity and leukemia stem cell properties [70,71]. Knockout of pyruvate kinase M2 (PKM2) enforces a metabolic shift toward mitochondrial respiration, which significantly impairs leukemia initiation and maintenance in vivo, providing direct evidence that blocking lactate production redirects pyruvate toward oxidative metabolism and inhibits leukemogenesis [23] and the leukemic cells’ survival. In FLT3 ITD^+^ AML cells, under inhibition of mitochondrial electron transport chain complex II, exogenous 13C-labeled lactate is converted to pyruvate and incorporated into TCA cycle intermediates, including citrate and succinate, indicating that AML cells can utilize lactate as an alternative carbon source to fuel mitochondrial metabolism when glycolysis-derived pyruvate entry is restricted [42]. Forte et al. [48] studied the contribution of nestin+ BMSCs to MLL-AF9-driven AML development and resistance to conventional chemotherapy in vivo. They showed that the metabolic profile of chemotherapy-treated blasts in liquid coculture with tridimensional mesenchymal stem cell aggregates (mesenspheres), showed 2–3-fold higher levels of TCA cycle intermediates fumarate, α-ketoglutarate (α-KG), and malate, thus underlining the relevance of TCA activation in AML development.

### 3.2. OXPHOS and Mitochondrial Dependence in HSCs, LSCs and AML Blasts

Legadinou et al. isolated sorted primary human AML CD34^+^CD38^−^ cells (LSC compartment) according to intracellular ROS levels, distinguishing ROS-low LSCs and ROS-high LSCs, and tested their functional capacity in xenotransplantation assays and metabolic features [38]: ROS-Low LSCs, more quiescent and enriched for stemness transcriptional programs, had significantly higher leukemia-initiating capacity in immunodeficient NSG mice, whereas ROS-High LSCs showed markedly reduced engraftment potential and increased mitochondrial activity and cell-cycle entry.

ROS-low LSCs exhibited elevated oxygen consumption rate (OCR) and active OXPHOS despite maintaining low ROS levels, and expressed higher BCL-2 levels. Treatment with the BCL-2 inhibitor ABT-737 not only impaired xenograft leukemia-initiating capacity but also induced collapse of the mitochondrial membrane potential, thereby compromising mitochondrial respiration and bioenergetic capacity. Jones et al. [39] extended these findings by revealing that ROS-low LSCs depend predominantly on AA metabolism to fuel the TCA cycle. Pharmacological blockade of AA metabolism selectively disrupts mitochondrial respiration and viability in ROS-low LSCs, sparing more differentiated leukemic blasts, which rely less on this metabolic axis. This establishes a direct mechanistic link between AA–driven TCA cycle activity, BCL-2–mediated mitochondrial stability, and LSC survival. Interestingly, Farge et al. [40] demonstrated that cytarabine-treatment-resistant AML cells display an increased reliance on oxidative metabolism characterized by elevated mitochondrial mass, membrane potential, and fatty acid oxidation. However, unlike ROS-low LSCs, these chemotherapy-resistant cells are not enriched for functional leukemia stem cell properties and represent an adaptive metabolic state rather than an intrinsically stem-like phenotype.

While this dual-state model provides a useful framework, accumulating evidence indicates that AML cells are not restricted to fixed metabolic phenotypes [2,52]. The dependence of LSCs on the TCA-OXPHOS axis is also shown by Amaia ML et al.’s demonstration that inhibition of the STAT3–MYC–SLC1A5 axis reduces intracellular levels of glutamine, glutathione, and multiple TCA cycle metabolites. This leads to reduced TCA cycle activity, inhibition of OXPHOS, and cell death in LSCs derived from newly diagnosed and relapsed patients, while sparing normal hematopoietic cells [72].

HSCs reside in the bone marrow niche and rely on anaerobic glycolysis for energy production; OXPHOS remains limited during the quiescent state [28,73]. This metabolic setup is critical for maintaining their quiescence, self-renewal, and long-term regenerative capacity [74]. HSC dormancy depends on PDK-mediated inhibition of pyruvate dehydrogenase (PDH), which restricts pyruvate conversion to acetyl-CoA and limits its entry into the TCA cycle, reducing OXPHOS; PDK2 and PDK4 loss causes defective maintenance of quiescence and transplantation capacity [75]. Concomitantly, transcriptional adaptations contribute to HSC quiescence; for example, MEIS1 has been shown to upregulate HIF-1α expression, thereby favoring glycolytic metabolism [28,76], and increased expression of the steroid receptor coactivator 3 (SRC-3). Hu M. et al. showed that SRC-3-deficient HSCs are less quiescent, more proliferative, and have cell homeostasis completely altered [77]. In contrast, progression toward the generation of more mature cells requires higher levels of OXPHOS [78,79]. Thus, HSCs exiting quiescence exhibit increased OXPHOS activity and consequently higher ROS generation. These ROS levels are regulated by multiple mechanisms, including increased expression of antioxidant enzymes such as superoxide dismutase (SOD) and microsomal glutathione transferase 1 (Mgst1) [80,81,82]. In addition, redirecting glucose-derived metabolites toward the PPP, rather than the TCA cycle, limits ROS production [83,84,85].

OXPHOS is a major source of ROS, which, per se, favors myeloid lineage differentiation of HSCs [86]. Even within the quiescent HSC, distinct metabolic profiles have been described: cells with relatively higher mitochondrial membrane potential—often referred to as “primed” HSCs—exhibit increased metabolic flux and a greater reliance on oxidative metabolism compared with deeply quiescent HSCs, consistent with the notion that metabolic activation precedes proliferative and differentiation responses [87,88].

Compared to normal hematopoietic cells, AML blasts demonstrate intrinsic dysfunction in OXPHOS [89]. Nevertheless, they retain a partial and context-dependent reliance on mitochondrial metabolism to sustain their bioenergetic and biosynthetic demands [40,90,91,92]. This reflects the functional heterogeneity of the blast population, with subsets displaying differential engagement of glycolytic and oxidative pathways. As a result, although OXPHOS may operate with reduced efficiency, it remains a relevant metabolic node contributing to leukemic cell survival and chemoresistance [36,40,42,93,94,95,96,97,98,99]. Sriskanthadevan S. et al. have shown that AML blasts exhibit reduced electron flux, reflected in a diminished spare respiratory capacity, which renders them highly sensitive to OXPHOS efficiency. In contrast, HSCs can compensate for energy deficits by upregulating glycolysis and electron transport flux [100].

MCL1 has emerged as a critical determinant of leukemic development and maintenance. Beyond its anti-apoptotic role, MCL1 regulates OXPHOS, respiratory function and metabolic fitness in AML blasts. Catalano et al. [101] demonstrated that primary AML blasts, as compared with normal HSCs, are characterized by higher basal OXPHOS; notably, this metabolic profile was associated with shorter overall survival and significant overexpression of MCL1. HK2 normally binds Voltage-dependent anion channel (VDAC), but in AML blasts this interaction is stabilized by MCL1, thereby promoting functional coupling between mitochondrial respiration and glycolysis. These findings provide a mechanistic insight and position MCL1 as a central integrator of mitochondrial integrity and metabolic plasticity in AML, linking enhanced OXPHOS activity, glycolytic support, and resistance to therapy.

### 3.3. Mitochondrial Plasticity and Therapy Resistance

Despite effective reduction in the bulk AML blast population with standard-of-care therapies, LSCs, different in their ability to regenerate leukemia, their relative quiescence, distinct immunophenotypic features, their association with poor clinical prognosis, and their unique metabolic programs [102,103], remain treatment-resistant and are associated with an increased risk of relapse [104].

Metabolomic profiling of LSCs and bulk AML blasts from primary patients, using mass spectrometry, identified 39 metabolites significantly enriched in LSCs, among them: citrate, α-ketoglutarate, and five glutathione-related metabolites, indicating enhanced mitochondrial oxidative metabolism in LSCs [38]. Proteome analysis comparing LSCs to AML leukemic blasts and healthy Hematopoietic stem and progenitor cells (HSPCs) provided strong evidence that LSCs harbor a characteristic energy metabolism, other than adhesion molecule composition and RNA-processing properties [105].

LSCs, differently from bulk AML blasts and HSCs, rely on OXPHOS for survival and energy production; OXPHOS inhibition selectively impacts LSC viability [38]. LSCs have been shown to acquire functional mitochondria from bone marrow stromal cells through direct intercellular transfer mechanisms, enhancing their oxidative capacity and resistance to chemotherapy; this phenomenon has been observed both in vitro and in vivo and mediated by endocytic pathways or gap junction proteins such as Connexin-43 [106,107]. Mechanistic studies implicate specific regulatory axes, such as STAT3–MYC, in controlling OXPHOS activity in LSCs derived from primary human AML specimens, and modulation of these pathways disrupts LSC metabolic homeostasis and survival [72]. The observation that LSCs rely on OXPHOS and fail to reactivate glycolysis upon OXPHOS inhibition renders it particularly susceptible to targeted interventions [38].

### 3.4. Mitochondrial Quality Control and Redox Homeostasis

**Figure 3:**
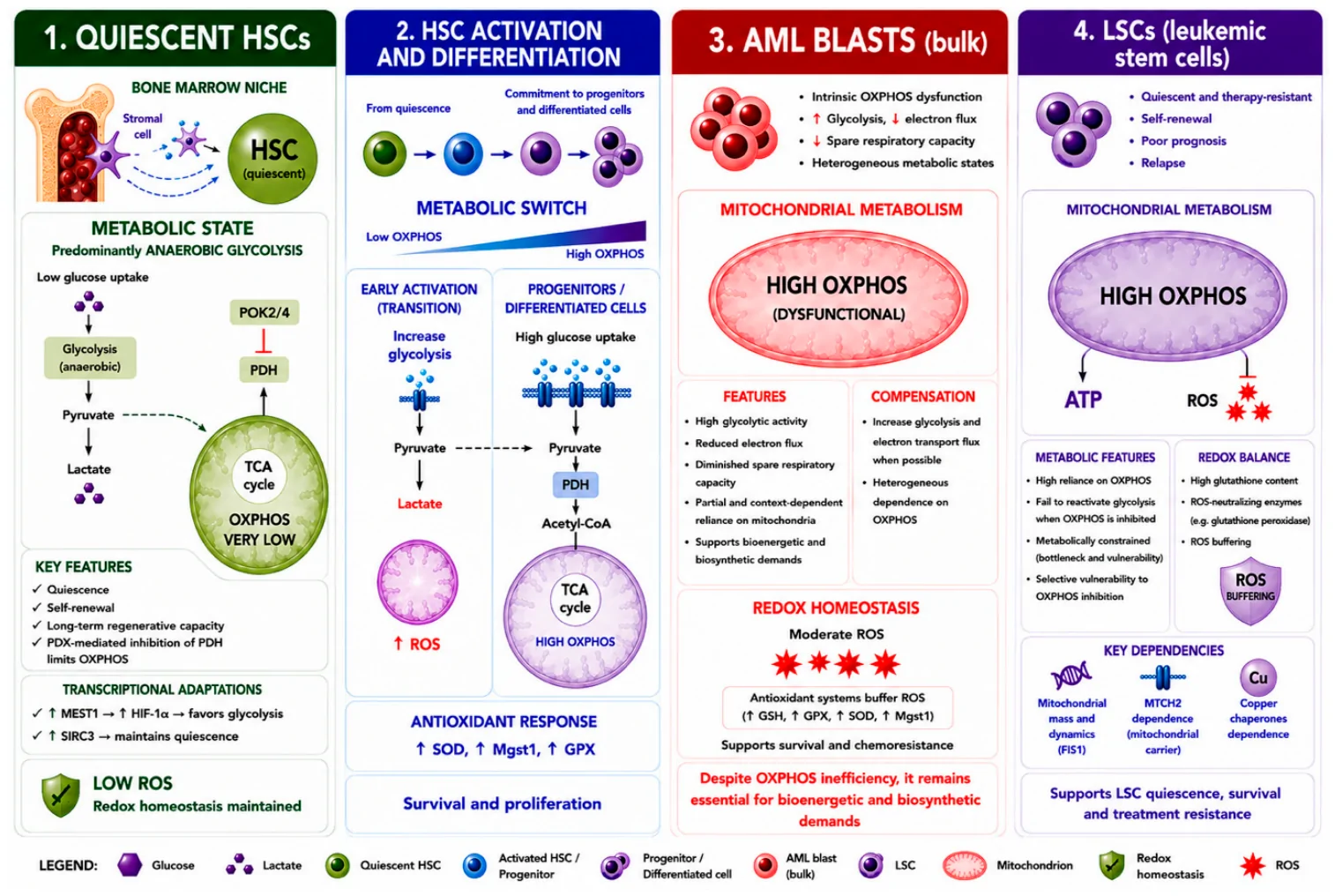
Metabolic adaptation from quiescent HSCs to AML blasts and leukemic stem cells (LSCs). Quiescent HSCs rely predominantly on anaerobic glycolysis and maintain low OXPHOS. HSC activation and differentiation involve a metabolic switch toward increased glucose uptake and mitochondrial OXPHOS. AML blasts display heterogeneous, often elevated OXPHOS with compensatory glycolysis and moderate ROS levels. LSCs are characterized by high OXPHOS, increased ATP production and redox buffering, supporting quiescence, self-renewal and therapy resistance. Arrows indicate: ↑ Increase, ↓ Decrease, and ⊣ Inhibition.

LSCs show: reduced mitochondrial mass, related to enhanced mitochondrial fission and autophagy mediated by mitochondrial fission protein 1 (FIS1) [63]; a dependence on mitochondrial carrier homolog 2 (MTCH2) [108]; and a strong dependence on mitochondrial copper distribution [109]. However, the elevated OXPHOS activity does not lead to high intracellular ROS levels, because antioxidant systems, including high glutathione content and ROS-metabolizing enzymes (glutathione peroxidase), effectively buffer ROS, maintaining redox homeostasis and supporting LSCs quiescence and survival [110,111] (Figure 3).

## 4. Amino Acid Metabolism in HSCs, LSCs and AML Blasts

### 4.1. Essential Amino Acids: BCAAs, Methionine and Tryptophan Metabolism

AML and normal cells differ in their utilization of amino acids (AA): they are essential not only for protein synthesis but also for energy production, nucleotide biosynthesis, redox balance, and regulation of epigenetic programs including post-transcriptional protein modification. There are 20 AAs in total, classified as essential, requiring uptake, and non-essential, which can be synthesized by our cells. Several lines of evidence have demonstrated that AA metabolism is important not only for HSCs but also for the development and survival of LSCs and AML blasts. Among essential AAs, branched-chain AAs (BCAAs: leucine, isoleucine, and valine) seem to have a relevant involvement in normal and leukemic hematopoiesis. For instance, restricting valine uptake reduces the proliferation and survival of HSCs and, in murine models, conditions the bone marrow to allow donor cell engraftment [112,113]. BCAT1 (branched-chain AA transaminase1) is enriched and overexpressed specifically in LSCs relative to non-stem AML blast cells. This overexpression is linked to altered α-ketoglutarate homeostasis and epigenetic regulation in LSCs [114]. BCAT1 overexpression in leukemic cells depletes intracellular α-ketoglutarate (α-KG), a cofactor for ten-eleven translocation (TET) enzymes’ activity, thereby inhibiting DNA demethylation. Perturbations in α-KG levels have been shown to correlate with survival in AML patients, depending on the presence or absence of IDH1 (isocitrate dehydrogenase 1) and TET mutations [114]. Gabapentin, an anticonvulsant drug with partial inhibitory activity against BCAT1, effectively abrogated the proliferation of AML blasts [115].

Methionine is converted to S-adenosyl-methionine (SAM). SAM serves as a methyl donor for DNA and protein (histone) methylation reactions catalyzed by SAM-dependent methyltransferases, including histone and DNA methyltransferases [116]. Cunningham et al. showed that dietary methionine starvation delayed the progression of patient-derived xenografts [117].

The majority of dietary tryptophan is catabolized through the kynurenine pathway, whose initial step is catalyzed by indoleamine 2,3-dioxygenase. Indoleamine 2,3-dioxygenase and its downstream metabolite kynurenine show immunosuppressive properties in the context of cancer, where they contribute to the inhibition of effector T-cell activation and the suppression of natural killer cell function. Kynurenine is present in high concentrations in the serum of AML patients, and it has been identified as an independent predictor of reduced overall survival, supporting a role for this pathway in facilitating immune evasion by leukemic cells [118].

### 4.2. Non-Essential Amino Acids: Glutamine and Cysteine Metabolism

Among nonessential AA, Glutamine appears to play a role of primary importance. Relative to HSCs, leukemic blasts exhibit an increased uptake of glutamine to support their survival and proliferation. The SLC1A5 transporter mediates the uptake of glutamine, including in AML cells. Glutamine is subsequently converted into glutamate and α-KG via glutaminolysis [43]. Glutamine depletion via shRNA-mediated knockdown of SLC1A5 induced apoptosis in AML samples, whereas glutamine deprivation did not elicit apoptosis in normal HSCs, indicating that leukemic blasts are more dependent on glutamine metabolism. Glutamine deprivation impairs the TCA cycle and funnels reducing equivalents (like NADH and FADH2) into the electron transport chain. Glutamine scarcity results in a pronounced reduction in OCR, suggesting that it also compromises OXPHOS [119,120]. Jones et al. [39] also showed that ROS-low LSCs predominantly depend on AA metabolism to sustain OXPHOS and support survival. In contrast, LSCs from relapsed AML circumvent AA deprivation by upregulating fatty acid (FA) metabolism. GLS1 (glutaminase 1), an enzyme that catalyzes the conversion of glutamine to glutamate, knockdown has been shown to inhibit the proliferation of primary AML cells. Furthermore, the use of GLS1 inhibitor CB-839 disrupts glutathione synthesis in AML cells, resulting in elevated ROS levels and induction of apoptotic cell death. GLS1 inhibition also sensitizes AML cells to chemotherapeutic agents such as arsenic trioxide (ATO) [120,121]. 

The asparagine synthetase (ASNS) catalyzes the conversion of aspartate into asparagine and glutamine into glutamate. Asparaginase converts asparagine to aspartic acid and it has shown potent anti-leukemic activity and has been used for the treatment of patients with de novo and relapsed AML [121,122,123].

LSCs exhibit a specific dependence on AAs, including cysteine. Cysteine depletion results in reduced GSH levels and loss of glutathionylation of succinate dehydrogenase subunit A, a component of electron transport chain complex II. The consequent reduction in OXPHOS and adenosine triphosphate (ATP) production compromises LSC survival [39,74].

### 4.3. Arginine Metabolism

AML blasts lack argininosuccinate synthetase-1, rendering them auxotrophic for arginine [124]. Rapid depletion of arginine using BCT-100, a pegylated recombinant human arginase, results in reduced AML proliferation and diminished engraftment following transplantation in xenograft models. Furthermore, BCT-100 exhibits synergistic cytotoxic effects when combined with cytarabine in AML blasts isolated from patients [125].

**Figure 4:**
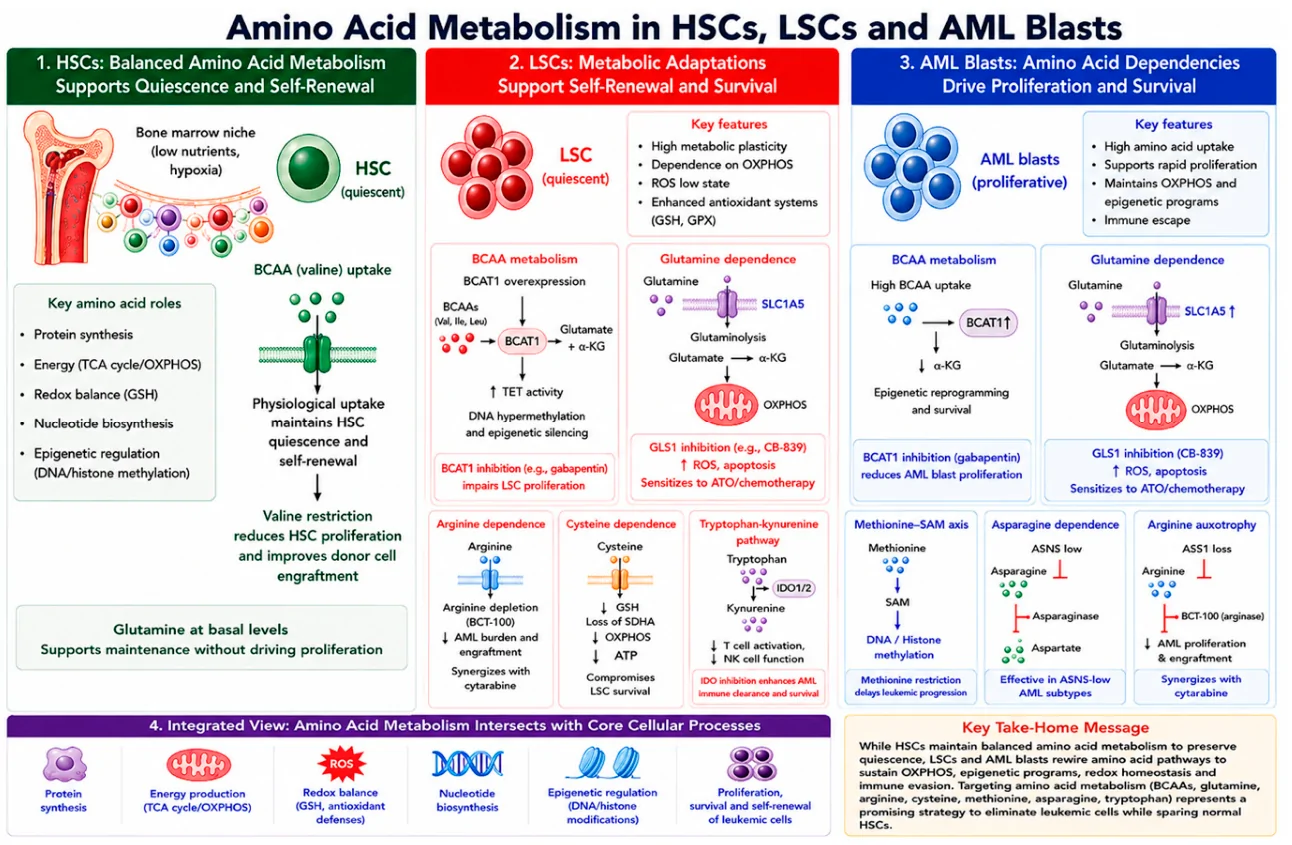
Amino acid metabolism in HSCs, LSCs and AML blasts. HSCs maintain a balanced amino acid metabolism supporting quiescence and self-renewal, whereas LSCs and AML blasts rewire amino acid utilization to sustain OXPHOS, redox homeostasis, epigenetic regulation and leukemic cell survival. These metabolic dependencies represent potential therapeutic vulnerabilities. BCAA, branched-chain amino acid (leucine, isoleucine, valine); BCAT1, branched-chain amino acid transaminase 1; α-KG, α-ketoglutarate; TET, ten-eleven translocation (DNA demethylases); GLS1, glutaminase 1; ASNS, asparagine synthetase; ASS1, argininosuccinate synthetase 1; IDO1/2, indoleamine 2,3-dioxygenase 1/2; OXPHOS, oxidative phosphorylation; ROS, reactive oxygen species; SAM, S-adenosylmethionine; GSH, glutathione; GPx, glutathione peroxidase; SDHA, succinate dehydrogenase subunit A; ATO, arsenic trioxide; NK, natural killer.

Intracellular arginine depletion reduces AML burden in patient-derived xenograft models, with enhanced efficacy observed in combination with cytarabine [126] (Figure 4).

## 5. Lipid Metabolism in HSCs, LSCs and AML Blasts

Fatty acid synthesis (FAS) and fatty acid oxidation (FAO) are dynamically regulated and functionally interconnected metabolic pathways. In HSCs, experimental studies have demonstrated that FAO is a critical metabolic mechanism for maintaining self-renewal capacity and regulating cell division. Ito et al. [127] identified a Promyelocytic Leukemia protein–Peroxisome Proliferator-Activated Receptor delta–dependent (PML–PPAR-δ) metabolic pathway regulating FAO in HSCs. Mechanistically, the PML–PPAR-δ–FAO pathway controls the asymmetric division of HSCs. Deletion of PPAR-δ or PML as well as inhibition of FAO, results in the symmetric commitment of HSC daughter cells, whereas PPAR-δ activation increases asymmetric cell division. This mechanism is essential for preserving stemness and preventing cellular exhaustion.

FAO in HSCs contributes not only to low-level ATP production but also to the generation of reducing equivalents to mitigate oxidative stress and potentially to metabolic signals that influence the stem cell epigenome [128]. Relative to normal HSCs, chemotherapy-resistant LSCs display increased dependence on FAO and OXPHOS. The enzyme VLCAD (very long-chain acyl-CoA dehydrogenase) is overexpressed in AML blasts, and its genetic or pharmacologic inhibition reduces mitochondrial respiration, ATP generation, proliferation, clonogenic capacity, and engraftment, demonstrating the functional importance of FAO in LSC biology [129].

FAS is downregulated in quiescent HSCs, switching to upregulated during progenitor expansion and differentiation to support anabolic growth and membrane biogenesis. Similarly, in AML, proliferating blasts engage FAS to support biomass accumulation and cell division, whereas LSCs preferentially rely on FAO-driven OXPHOS to maintain quiescence, survival, and chemoresistance [128,130].

Collectively, these data support a model in which FAO sustains stemness in normal HSCs and LSCs, but its functional role differs: in HSCs, it regulates cell fate and preserves quiescence, whereas in LSCs, it fuels high OXPHOS activity to promote survival and chemoresistance.

**Figure 5:**
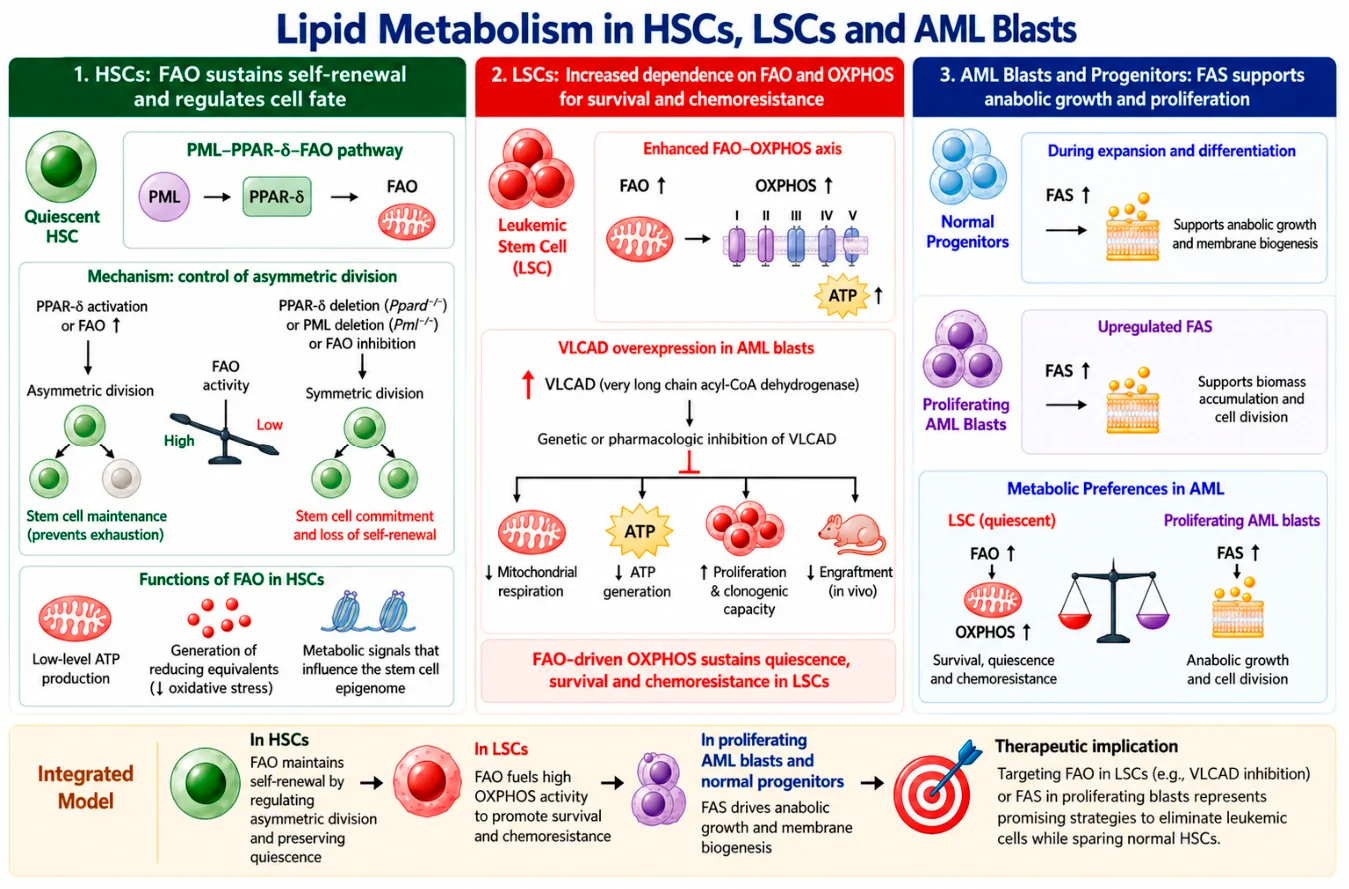
Lipid metabolism in HSCs, LSCs and AML blasts. FAO supports HSC quiescence and self-renewal, whereas LSCs rely on FAO-driven OXPHOS for survival and chemoresistance. In contrast, proliferating AML blasts preferentially increase FAS to sustain anabolic growth and proliferation. These distinct metabolic dependencies may provide selective therapeutic vulnerabilities. FAO, fatty acid oxidation; FAS, fatty acid synthesis; OXPHOS, oxidative phosphorylation; PML, promyelocytic leukemia protein; PPAR-δ, peroxisome proliferator-activated receptor delta; VLCAD, very long chain acyl-CoA dehydrogenase; ATP, adenosine triphosphate. Arrows indicate: ↑ Increase, ↓ Decrease, and ⊣ Inhibition.

FAS, in contrast, is associated with anabolic proliferation and differentiation in both normal progenitors and proliferating leukemic blasts (Figure 5).

## 6. Metabolism of APL

### 6.1. PML::RARA and APL Biology

Recent studies from our lab have clarified the metabolic dependency of acute promyelocytic leukemia (APL). Driven by the PML::RARA fusion protein, APL is characterized by a differentiation block at the promyelocyte stage. PML::RARA acts as a dominant-negative transcription factor through recruitment of transcriptional corepressors and histone deacetylase complexes, while disruption of PML impairs PML nuclear body function and tumor-suppressive pathways, including the PML–p53 axis [47,131,132]. The efficacy of APL therapy, all-trans retinoic acid (ATRA) and arsenic trioxide (ATO), relies on PML::RARA degradation, restoration of PML nuclear bodies function, and consequent induction of differentiation and apoptosis of leukemic cells [133,134].

### 6.2. Obesity and Metabolic Dysregulation in APL

Obesity is a well-recognized risk factor influencing both the incidence and clinical outcome of APL. Epidemiological evidence from large population-based and cross-sectional studies indicates that elevated BMI confers a greater risk of developing APL than non-APL AML. Moreover, obesity is associated with an increased frequency of FLT3 mutations and to the transcriptional activation of pathways involved in polyunsaturated fatty acid metabolism, supporting the concept that obesity-driven metabolic dysregulation contributes to APL pathogenesis. Clinical evidence also indicates that patients with APL tend to have a higher prevalence of overweight and obesity than patients with non-APL AML. Meta-analytic data show that high BMI is associated with worse overall survival (and a higher risk of differentiation syndrome in APL), whereas the prognostic impact of obesity is less clear in non-APL AML [135,136]. Although the mechanisms underlying this relationship remain incompletely understood, proposed explanations include obesity-related inflammatory cytokines, altered retinoid and lipid metabolism, insulin/IGF signaling, and adipocyte-leukemia cell interactions within the marrow niche. Thus, obesity and fat-related metabolism must play a specific role in APL biology and warrant further investigation into the interplay between metabolic dysregulation and PML::RARA-driven leukemogenesis.

### 6.3. Glucose, Lipid and Amino Acid Metabolic Rewiring in APL

We provided the first comprehensive metabolic characterization of APL [41] and demonstrated that PML::RARA expression profoundly rewires cellular metabolism. Compared with normal promyelocytes, APL cells exhibited markedly reduced glycolytic activity and a striking dependence on long-chain fatty acids (LCFAs) as the principal fuel source sustaining mitochondrial respiration. This metabolic phenotype was associated with increased levels of TCA cycle intermediates, elevated acylcarnitine concentrations, and overexpression of the carnitine transporter SLC22A16, collectively indicating a preferential utilization of lipid-derived substrates. These metabolic alterations exposed therapeutically exploitable vulnerabilities. Inhibition of CD36-mediated fatty acid uptake restored sensitivity to arsenic trioxide in resistant NB4 clones, supporting a role for FAO in treatment resistance. The suppression of glycolysis by PML::RARA also increases dependence on mitochondrial bioenergetics, providing a rationale for OXPHOS-targeting strategies, including venetoclax-based combinations. Thus, APL provides an example of lineage-specific metabolic rewiring in which PML::RARA hijacks metabolic programs that normally support stem-cell maintenance, including the PML–PPAR-δ–FAO axis described in normal hematopoiesis [127], These findings suggest that FAO-supported OXPHOS may contribute to therapeutic resistance in APL, as observed in other therapy-resistant AML populations [40]. Amino acid metabolism is also extensively rewired in APL. Metabolomic analyses [41] revealed significant perturbations of pathways linked to arginine biosynthesis and the urea cycle, including the accumulation of arginine and its metabolism intermediates that may contribute to mitochondrial function and anabolic requirements.

### 6.4. Arginine Metabolism and Anaplerosis in APL

The argininosuccinate shunt represents a metabolic connection between the urea cycle and the TCA cycle. Through the sequential activity of argininosuccinate synthetase (ASS1) and argininosuccinate lyase (ASL), citrulline and aspartate are converted into argininosuccinate and subsequently into arginine and fumarate. Fumarate can directly replenish the TCA cycle, thereby providing an anaplerotic source of carbon that supports mitochondrial respiration. Increased utilization of this pathway may therefore contribute to the maintenance of OXPHOS observed in PML::RARA-positive cells.

These findings are particularly noteworthy in light of previous studies demonstrating arginine auxotrophy in several AML subtypes due to reduced ASS1 expression [125,126]. In contrast, the metabolic profile of APL suggests that activation of the argininosuccinate pathway, together with enhanced FAO and reduced glycolytic flux, may contribute to mitochondrial metabolic adaptation and survival under therapeutic stress, although the functional activity of ASS1 and ASL in APL cells remains to be directly demonstrated.

APL may therefore represent a relatively constrained metabolic state compared with the broader metabolic plasticity observed in AML, potentially exposing specific mitochondrial vulnerabilities.

## 7. Metabolic Vulnerabilities as Therapeutic Targets

Based on the metabolic background depicted throughout this review, metabolism-directed therapies in AML can be grouped into five major categories: (i) targeting onco-metabolic enzymes and redox homeostasis; (ii) targeting mitochondrial metabolism and oxidative phosphorylation (OXPHOS); (iii) interfering with nutrient utilization; (iv) targeting lipid metabolism; and (v) modulating glucose metabolism and glycolytic pathways. Representative metabolism-targeting agents currently under clinical and preclinical development for AML are summarized in Table 2.

### 7.1. Targeting Onco-Metabolic Enzymes and Redox Homeostasis

Mutations in IDH1 and IDH2 represent a paradigm of oncometabolism in AML. Neomorphic IDH mutations promote the production of the oncometabolite D-2-hydroxyglutarate (2-HG), which inhibits α-KG-dependent dioxygenases, leading to DNA and histone hypermethylation, impaired differentiation, and leukemogenesis. Pharmacological inhibition of mutant IDH1/2 reduces 2-HG levels and promotes myeloid differentiation, leading to the clinical approval of ivosidenib and olutasidenib for IDH1-mutated AML and enasidenib for IDH2-mutated AML [137,138,139]. Thus, mutant IDH represents the first clinically validated metabolic target in AML and provides proof-of-concept for targeting additional metabolic vulnerabilities, including mitochondrial respiration, nutrient utilization, lipid metabolism, glycolysis, and redox homeostasis.

Targeting redox homeostasis has emerged as a promising metabolic strategy in AML. ATO induces mitochondrial ROS accumulation and oxidative stress-mediated apoptosis. ATO also suppresses glycolysis and impairs mitochondrial bioenergetics, further contributing to its antileukemic effects. Azacitidine enhances ATO activity by upregulating AQP9, increasing intracellular arsenic uptake. Another promising approach combines ATO with artemisinin derivatives, which further amplify oxidative stress by promoting iron-dependent ROS generation, lysosomal dysfunction, and mitochondrial damage. These combinations remain under preclinical and early clinical evaluation [140].

**Table 2:** Representative metabolism-targeting agents under clinical and preclinical development in AML.

Metabolic Pathway	Target	Representative Agent(s)	Clinical Development	NCT Clinical Trials	Main Adverse Effects	Ref.
Oncometabolism	Mutant IDH1	Ivosidenib	Approved (AML)	NCT07607418, NCT03245424	Differentiation syndrome, QT prolongation, leukocytosis, fatigue	[137,141]
	Mutant IDH1	Olutasidenib	Approved (AML)	NCT07130695, NCT07304011, NCT06543381	Differentiation syndrome, leukocytosis, transaminase elevation, nausea	[138]
	Mutant IDH2	Enasidenib	Approved (AML)	NCT04092179, NCT02632708 NCT03013998	Differentiation syndrome, indirect hyperbilirubinemia, nausea, decreased apetite	[137]
	Mutant IDH1/2	Vorasidenib	Approved (glioma)	NCT04603001	Fatigue, headache, nauseas, liver enzyme elevation	[142]
Redox Homeostasis	ROS induction	Arsenic trioxide (ATO)	Approved (APL)	NCT03031249, NCT03381781 NCT03855371	QT prolongation, differentiation syndrome, hepatotoxicity, electrolyte abnormalities	[140]
	ROS induction	Artesunate (ART)	Preclinical/Early clinical	NCT07022743	Gastrointestinal symptoms, transient transaminase elevation, hematologic toxicity	[143,144]
	Glutaminase 1 (GLS1)	Telaglenastat (CB-839)	Phase I/II	NCT02071927, NCT03047993	Nausea, fatigue, decreased appetite, gastrointestinal toxicity	[145]
	Mutant p53/GSH depletion	Eprenetapopt (APR-246)	Phase II/III	NCT03931291, NCT03072043	Neurologic symptoms, nausea, fatigue, cytopenias	[146,147]
Mitochondrial metabolism/OXPHOS	BCL-2	Venetoclax + azacitidine	Approved	NCT05001828, NCT04912063 NCT05144243, NCT07044687, NCT03466294	Neutropenia, thrombocytopenia, febrile neutropenia, infections	[148,149,150]
	MCL1	AMG176; AMG397; AZD5991; S64315 (MIK665); PRT1419	Phase I	NCT03797261, NCT02675452, NCT03465540, NCT03218683, NCT04702425, NCT02979366, NCT04543305	Cardiac toxicity, Gastrointestinal toxicity, transaminase elevation.	[151,152,153]
	MCL1	PROTAC degraders	Preclinical		Not established	[154]
	ETC Complex I	IACS-010759	Phase I	NCT02882321	Lactic acidosis/hyperlactatemia, neurotoxicity, fatigue	[155]
	AMPK	Metformin	Preclinical/Early clinical	NCT01849276, NCT05854966, NCT06537843	Gastrointestinal symptoms, diarrhea, nausea; rare lactic acidosis	[156]
	AMPK	Buformin	Preclinical	-	Not established, potential lactic acidosis	[157]
	PDH/α-KGDH	Devimistat (CPI-613)	Phase III completed	NCT03504410, NCT01520805, NCT05854966, NCT01034475	Cytopenias, gastrointestinal toxicity, fatigue, transaminase elevation	[158]
Nutrient utilization	Arginine depletion	BCT-100	Phase I/II	NCT02899286, NCT03455140	Nausea, fatigue, thrombocytopenia	[125]
	Arginine depletion	ADI-PEG20	Phase I/II	NCT05001828, NCT02875093, NCT01910012	Fatigue, nausea, hypersensitivity, injection-site reactions	[159]
	Asparagine depletion	L-asparaginase	Approved (ALL); evaluated in AML	NCT04326439, NCT02283190, NCT01810705	Hypersensitivity, pancreatitis, thrombosis, hepatotoxicity	[160,161]
	Asparagine depletion	Pegaspargase	Approved (ALL); evaluated in AML	NCT05521087, NCT03860844	Hypersensitivity, pancreatitis, thrombosis, hepatotoxicity	[162]
	IDO1 (Tryp depletion)	Epacadostat	Phase I/II	NCT03444649, NCT03491579, NCT01822691	Fatigue, nausea, diarrhea, headache	[118,163]
Lipid metabolism	CD36	SMS121	Preclinical	-	Not established	[164]
	CPT1	Etomoxir; ST1326; Avocatin B	Preclinical	-	Not established	[165,166]
	VLCAD	VLCAD inhibitors	Preclinical	-	Not established	[129]
	FASN	Denifanstat (TVB-2640)	Clinical (non-AML)	NCT04352361, NCT03808558	Fatigue, nausea, gastrointestinal toxicity, transaminase elevation	[167]
	HMG-CoA reductase	Pitavastatin	Phase I (AML combinations)	NCT04512105	Myalgia, elevated creatine kinase, hepatotoxicity	[168]
Glucose metabolism	MCT1	AZD3965	Phase I (Advanced cancer)	NCT01791595	Hyperlactatemia, gastrointestinal symptoms, fatigue	[169]
	AKT	MK-2206	Phase I/II	NCT01253447, NCT01231919	Hyperglycemia, rash, QTc prolongation	[170,171]
	mTOR	Everolimus	Phase I/II	NCT01074086, NCT02539459	Mucositis, cytopenias, infections, hyperglycemia	[172]
	mTOR	Temsirolimus	Phase I/II	NCT01611116, NCT01184898	Stomatitis, rash, cytopenias, hyperglycemia	[173]

ADI-PEG20, pegylated arginine deiminase; AKT, protein kinase B; ALL, acute lymphoblastic leukemia; AML, acute myeloid leukemia; AMPK, AMP-activated protein kinase; APL, acute promyelocytic leukemia; ART, artesunate; ATO, arsenic trioxide; BCL-2, B-cell lymphoma 2; CPT1, carnitine palmitoyltransferase 1; ETC, electron transport chain; FASN, fatty acid synthase; GLS1, glutaminase 1; GSH, glutathione; HMG-CoA, 3-hydroxy-3-methylglutaryl-coenzyme A; IDH, isocitrate dehydrogenase; IDO1, indoleamine 2,3-dioxygenase 1; MCL1, myeloid cell leukemia 1; MCT1, monocarboxylate transporter 1; mTOR, mechanistic target of rapamycin; OXPHOS, oxidative phosphorylation; PDH, pyruvate dehydrogenase; α-KGDH, α-ketoglutarate dehydrogenase; PROTAC, proteolysis-targeting chimera; ROS, reactive oxygen species; VLCAD, very-long-chain acyl-CoA dehydrogenase.

A complementary strategy targets glutamine metabolism through inhibition of glutaminase. Telaglenastat (CB-839), the first glutaminase inhibitor to enter clinical development, impairs glutathione biosynthesis, increases intracellular ROS, and disrupts mitochondrial metabolism. Preclinical studies have demonstrated synergistic activity with venetoclax, azacitidine, and FLT3 inhibitors, supporting the ongoing clinical evaluation of glutaminase inhibition in AML [145].

Eprenetapopt (APR-246), a mutant p53 reactivator currently under clinical investigation, also disrupts redox homeostasis by depleting intracellular glutathione and increasing ROS accumulation, sensitizing AML cells to ferroptosis and providing a rationale for combination therapies with hypomethylating agents and venetoclax [146,147]. Future strategies will likely combine redox-disrupting agents with inhibitors of mitochondrial metabolism or anti-apoptotic pathways to overcome metabolic adaptation and therapeutic resistance.

### 7.2. Mitochondrial Metabolism and OXPHOS

Mitochondrial oxidative phosphorylation (OXPHOS) is a major therapeutic vulnerability in AML, particularly in LSCs and therapy-resistant cells. The most clinically successful strategy targeting mitochondrial dependence is venetoclax combined with azacitidine (Aza-Ven), now standard first-line therapy for older or unfit patients with AML [148,149,174]. Beyond its pro-apoptotic activity, venetoclax disrupts AA-driven OXPHOS in LSCs, reducing mitochondrial respiration and ATP production. However, resistant cells restore OXPHOS by increasing fatty acid oxidation and alternative fuel utilization, frequently accompanied by upregulation of MCL1 and BCL-XL [174,175]. Monocytic differentiation further promotes a shift from BCL-2 to MCL1 dependence [176], preserving mitochondrial fitness and survival despite BCL-2 inhibition.

MCL1 has emerged as a particularly attractive therapeutic target because its functions extend well beyond apoptosis regulation. MCL1 promotes long-chain fatty acid oxidation through interaction with Acyl-CoA synthetase long-chain family member 1 (ACSL1), further linking this anti-apoptotic protein to mitochondrial fuel utilization [151], MCL1 integrates redox signaling with venetoclax resistance, as intracellular superoxide promotes AKT-dependent phosphorylation of MCL1 at Thr163, increasing its stability by preventing ubiquitination and proteasomal degradation [177]. These findings identify MCL1 as a central regulator of mitochondrial metabolism, bioenergetic plasticity, and therapeutic resistance in AML.

**Figure 6:**
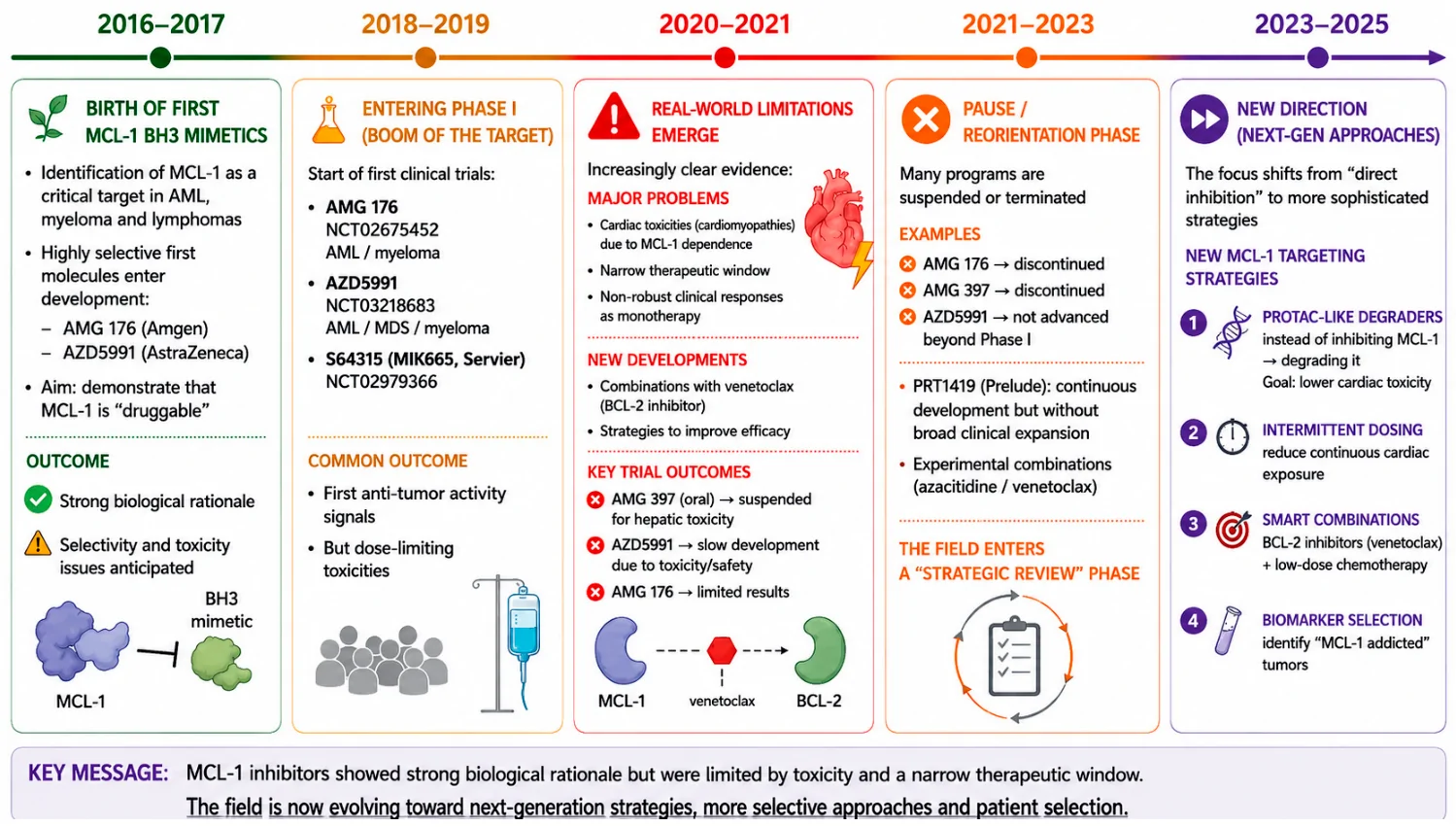
Evolution of MCL1-targeted therapies in AML. The figure summarizes the clinical development of direct MCL1 inhibitors, from early biological rationale and first-in-human trials to the emergence of dose-limiting toxicities and a narrow therapeutic window. These limitations have redirected the field toward next-generation approaches, including targeted protein degradation, intermittent dosing, rational combinations, and biomarker-guided patient selection.

The strong biological rationale prompted the development of selective MCL1-directed BH3 mimetics [152], but clinical development has been hindered by a narrow therapeutic window and limited single-agent efficacy, with cardiac toxicity being the principal obstacle to sustained systemic inhibition [152,153]. Consequently, current efforts are shifting toward more selective strategies, including intermittent dosing schedules, biomarker-guided patient selection, rational combination therapies, and targeted protein degradation. In particular, PROTAC-based degraders recruit E3 ubiquitin ligases to promote MCL1 ubiquitination and proteasomal degradation, offering the potential to achieve effective target suppression at lower systemic exposure and with improved tolerability [154] (Figure 6).

Direct inhibition of the mitochondrial ETC represents another attractive therapeutic strategy. IACS-010759, a selective inhibitor of mitochondrial Complex I (NADH dehydrogenase), suppresses oxygen consumption, ATP generation, and mitochondrial respiration, preferentially targeting AML cells with high OXPHOS activity [155]. However, OXPHOS is also essential for normal tissues, particularly highly energy-demanding organs such as the brain and heart. Thus, therapeutic development will require careful assessment of on-target toxicity and a sufficient therapeutic window [155]. Although preclinical studies demonstrated potent activity against primary AML samples and LSCs, dose-limiting neurotoxicity has restricted the clinical development of OXPHOS inhibitors, highlighting the challenge of targeting mitochondrial metabolism in tissues with high energetic demands [155,178]. Notably, additional approaches aim to interfere with upstream regulators of mitochondrial function. AMPK, the master cellular energy sensor, coordinates mitochondrial biogenesis, oxidative metabolism, and metabolic adaptation under energetic stress. AML cells exploit this pathway to preserve mitochondrial integrity, glucose utilization, and FIS1-dependent mitophagy, thereby sustaining LSC self-renewal [62,63]. Consequently, pharmacological modulation of AMPK, including with the biguanides metformin and buformin, has been explored as a strategy to impair mitochondrial metabolism and sensitize leukemic cells to OXPHOS inhibition [156,157]. Beyond AMPK modulation, metformin has also been shown to induce ferroptosis associated with lipidomic remodeling in AML cells. Although encouraging preclinical data have been reported, the therapeutic role of AMPK-targeting strategies in AML remains to be established [156].

A complementary approach targets the TCA cycle upstream of the ETC. Devimistat (CPI-613), a lipoate analog, inhibits PDH and α-ketoglutarate dehydrogenase (α-KGDH), thereby restricting mitochondrial carbon flux and oxidative metabolism. Encouraging early-phase results in relapsed/refractory AML led to the randomized phase III ARMADA trial evaluating devimistat in combination with high-dose cytarabine and mitoxantrone. Although the study failed to improve remission rates or overall survival, devimistat remains an important clinical proof-of-concept that TCA-cycle metabolism is a therapeutically targetable vulnerability in AML [158].

Collectively, these studies establish mitochondrial metabolism as a major therapeutic vulnerability in AML. However, because many metabolic pathways are also essential for normal tissues, particularly the heart and brain, therapeutic development will require careful assessment of on-target toxicity and a sufficient therapeutic window. Current strategies target multiple components of mitochondrial function, including BCL-2 and MCL1-dependent survival, electron transport chain activity, TCA-cycle metabolism, and mitochondrial quality control. Future clinical benefits will likely rely on biomarker-guided combination therapies that overcome metabolic plasticity and selectively eradicate OXPHOS-dependent LSCs.

### 7.3. Therapies Interfering with Nutrient Utilization

Targeting nutrient utilization has emerged as another promising metabolic strategy in AML, exploiting the dependence of leukemic cells on specific AAs required for mitochondrial metabolism, biosynthesis, and redox homeostasis. Among nutrient-targeting strategies, glutaminase inhibition with telaglenastat (CB-839) represents the most advanced approach and has been discussed in the previous section because of its dual effects on glutamine metabolism and redox homeostasis [145]. Arginine depletion represents another clinically relevant strategy: because a subset of AMLs exhibits reduced expression of argininosuccinate synthetase-1 (ASS1), leukemic cells become dependent on extracellular arginine. Arginine-degrading enzymes, including pegylated arginase (BCT-100) and pegylated arginine deiminase (ADI-PEG20), have shown encouraging preclinical activity and are currently being investigated in early-phase clinical studies, particularly in combination with chemotherapy or venetoclax-based regimens [125,126,159].

Another clinically established approach exploits asparagine auxotrophy. L-asparaginase and pegaspargase, primarily used in acute lymphoblastic leukemia, have also demonstrated antileukemic activity in selected AML subsets, particularly those with low asparagine synthetase (ASNS) expression, supporting their evaluation in biomarker-selected patients [122,123,162].

Targeting tryptophan metabolism has also emerged as a promising therapeutic strategy. Inhibitors of IDO1 (e.g., epacadostat) have reached phase III clinical trials in solid tumors, while in hematologic malignancies they are currently being evaluated in phase I/II studies in AML and MDS, mainly in combination with hypomethylating agents or immunotherapies. Although clinical efficacy remains to be established, these studies support IDO1 as a promising therapeutic target for combination strategies aimed at overcoming immune escape and treatment resistance [118,163].

In contrast, therapeutic targeting of other nutrient pathways, including methionine, branched-chain AA, and cysteine metabolism, remains largely preclinical despite strong biological rationale. Future strategies will likely combine AA depletion with inhibitors of mitochondrial metabolism or anti-apoptotic pathways to overcome metabolic adaptation and improve therapeutic efficacy.

### 7.4. Compounds Targeting Lipid Metabolism

Leukemic cells exploit both exogenous fatty acid uptake and endogenous lipid synthesis, while therapy-resistant leukemic stem cells (LSCs) increasingly depend on fatty acid oxidation (FAO) to sustain OXPHOS and survival. Accordingly, therapeutic strategies are being developed to interfere with three major steps of lipid metabolism: fatty acid uptake, mitochondrial FAO, and de novo fatty acid synthesis (FAS) [165,179,180].

Fatty acid uptake can be targeted through inhibition of CD36, a major membrane transporter that facilitates the acquisition of extracellular lipids from the bone marrow microenvironment. CD36 expression has been associated with enhanced lipid utilization, leukemic survival, and therapy resistance [180,181]. In preclinical studies, CD36 inhibition, including with the small-molecule inhibitor SMS121, reduced fatty acid uptake and impaired AML cell viability [164]. CD36-dependent lipid metabolism has been implicated in immune escape and resistance to hypomethylating agents, supporting the potential of targeting lipid acquisition at the interface between leukemic cells and their microenvironment [182].

Pharmacological inhibition of CPT1, the rate-limiting enzyme controlling mitochondrial fatty acid entry, with compounds such as etomoxir, ST1326, or avocatin B has shown antileukemic activity in preclinical models. However, the clinical translation of direct FAO inhibitors has been limited by off-target effects, toxicity, and the physiological importance of fatty acid metabolism in normal tissues [165,166].

An alternative approach targets very long-chain acyl-CoA dehydrogenase (VLCAD), the enzyme catalyzing the first step of mitochondrial β-oxidation. VLCAD is overexpressed in subsets of AML, where it supports FAO, OXPHOS, and leukemic cell survival, making it a promising alternative target [129].

AML blasts can activate de novo fatty acid synthesis to provide lipids for membrane biogenesis, signaling, and redox adaptation. This pathway can be targeted at key enzymatic nodes, including acetyl-CoA carboxylase (ACC), fatty acid synthase (FASN), and stearoyl-CoA desaturase (SCD) [175,179]. FASN inhibitors reduce lipid synthesis and leukemic growth in preclinical models, whereas inhibition of fatty acid desaturation may be particularly relevant in relapsed AML, where increased lipid remodeling contributes to metabolic adaptation and Aza-Ven resistance [179,183]. Although selective FASN inhibitors such as denifanstat (TVB-2640) have reached clinical-stage development in other diseases and solid tumors, lipid-synthesis inhibitors remain predominantly preclinical in AML [167,184].

Collectively, these findings establish lipid metabolism as a central metabolic vulnerability in AML. Although no lipid-targeted therapy has yet been approved for clinical use in AML, inhibitors of CD36, CPT1, VLCAD, and FASN continue to show encouraging preclinical activity. Among lipid-targeting strategies, inhibition of the mevalonate pathway has recently gained considerable attention. Statins reduce cholesterol biosynthesis and the production of isoprenoid intermediates required for protein prenylation, thereby affecting multiple signaling pathways involved in leukemic cell survival. Pitavastatin has shown synergistic antileukemic activity in combination with venetoclax by further impairing mitochondrial metabolism and is currently being evaluated in clinical trials for patients with AML [168]. Future clinical translation will require biomarker-driven identification of lipid-dependent AML subsets and rational combination therapies to prevent compensatory metabolic rewiring.

### 7.5. Agents Modulating Glucose Metabolism and Glycolytic Pathways

Direct inhibition of glycolysis has achieved limited clinical translation in AML [48,162]. Among promising strategies is targeting lactate transport through inhibition of monocarboxylate transporter 1 (MCT1). The selective MCT1 inhibitor AZD3965 has reached phase I clinical evaluation in patients with advanced solid tumors and aggressive B-cell lymphomas, demonstrating target engagement and an acceptable safety profile [169]. Although AZD3965 has not yet been evaluated clinically in AML, its mechanism of disrupting lactate transport and glycolytic metabolism provides a rationale for future investigation in glycolysis-dependent AML subsets.

In addition to these approaches, glucose metabolism can be indirectly targeted by inhibiting the PI3K/AKT/mTOR pathway, a central regulator of glycolytic gene expression. Inhibition of this pathway downregulates GLUT1, HK2, and LDHA, thereby reducing glycolytic flux and reprogramming glucose metabolism in AML cells [185]. Clinically, the AKT inhibitor MK-2206 and the mTOR inhibitors everolimus and temsirolimus have shown limited efficacy as single agents but remain attractive candidates for combination regimens with chemotherapy or other metabolism-targeting agents [170,171].

### 7.6. Metabolic Adaptation to Therapy and Effects on Normal and Immune Cells

Therapy can reshape AML metabolism and expose or select specific metabolic dependencies. Venetoclax-based treatment suppresses amino-acid metabolism, TCA-cycle activity and OXPHOS in LSCs, whereas venetoclax-resistant cells can adapt by increasing fatty-acid oxidation and alternative pathways sustaining mitochondrial function [39,48,176]. Importantly, these vulnerabilities are not absolute, as normal HSCs and hematopoietic cells retain greater metabolic flexibility, although targeted metabolic pathways are also required for normal cell function [20,23,24,25]. Metabolic effects should also be considered in the immune compartment: resting T and NK cells have relatively low metabolic requirements, whereas activation induces increased glycolysis and OXPHOS to support proliferation and effector functions [37,186]. In AML, nutrient competition and metabolites such as lactate and kynurenine can further impair T- and NK-cell metabolism and function [186,187]. Thus, metabolic differences between AML and normal cells remain therapeutically relevant, but therapy-induced metabolic plasticity and effects on normal hematopoietic and immune cells must be considered when defining metabolic vulnerabilities and therapeutic windows.

## 8. Future Directions

Despite significant advances in understanding AML metabolism, translating metabolic vulnerabilities into effective therapies remains challenging. The remarkable metabolic plasticity of leukemic cells enables rapid compensation following inhibition of individual pathways, highlighting the need for rational combination strategies rather than single-agent metabolic interventions. Future therapeutic approaches will likely combine inhibitors of mitochondrial function, nutrient utilization, lipid metabolism, or redox homeostasis with established treatments such as venetoclax-based regimens, hypomethylating agents, targeted therapies, and immunotherapies.

An important challenge will be the identification of biomarkers capable of stratifying patients according to their functional metabolic dependencies and plasticity. Integrating functional metabolic profiling with genomic, transcriptomic, proteomic, and metabolomic analyses may better identify actionable vulnerabilities and potential metabolic escape routes than static metabolic measurements alone, thereby supporting biomarker-guided combination therapies.

Finally, targeting metabolic adaptations that sustain leukemic stem cells and therapeutic resistance represents one of the most promising strategies to improve long-term disease control. A deeper understanding of metabolic crosstalk between leukemic cells and the bone marrow microenvironment, is expected to accelerate the clinical translation of metabolism-based therapies in AML. Importantly, metabolic interventions may also affect antileukemic immune responses. T cells and NK cells rely on specific metabolic programs for proliferation and cytotoxic function, while AML cells can reshape the bone marrow metabolic environment through nutrient competition and accumulation of metabolites such as lactate and kynurenine, thereby impairing immune-cell function [25,186,187]. Conversely, targeting metabolic pathways such as arginine metabolism may enhance T-cell and CAR-T-cell activity against AML [188]. Thus, the effects of metabolism-targeted therapies on immune-cell fitness and their potential interactions with immunotherapies should be considered when defining therapeutic strategies.

## Figures and Tables

**Table 1:** Genetic background, differentiation state and metabolic dependencies across the AML continuum.

Genetic/Biological Context	Differentiation or Cellular State	Main Metabolic Features and Dependencies	References
Normal HSC	Quiescent, self-renewing	Predominantly glycolytic metabolism, low OXPHOS and controlled ROS production, supporting quiescence and long-term stem-cell maintenance	[20,21,22,23,24,25,27,28,29]
Normal hematopoietic progenitors	Proliferating and differentiating	Increased metabolic activity and mitochondrial OXPHOS during differentiation and lineage commitment	[20,21,22,23,24,25,27,28,29]
AML, LSC-enriched ROS-low populations	Quiescent or stem-like, leukemia-initiating	Dependence on OXPHOS, amino-acid metabolism and TCA-cycle activity; BCL-2-associated mitochondrial maintenance supports survival	[38,39]
AML, bulk leukemic blasts	Predominantly proliferative, with heterogeneous differentiation states	Variable glycolysis and pentose phosphate pathway activity, with heterogeneous OXPHOS and substrate utilization	[2,24,30,31,32,33,34]
FLT3-ITD-mutated AML	Genetically defined leukemic blasts	Glutamine metabolism contributes to leukemic-cell survival; lactate can provide an alternative carbon source for TCA-cycle intermediates under mitochondrial metabolic stress	[42,43]
NPM1-mutated AML (NPM1c+)	Genetically defined AML	NPM1c+ can disrupt PTEN regulation and promote PI3K/AKT signaling, linking oncogenic signaling to cellular survival and metabolic regulation	[44]
IDH1/IDH2-mutated AML	Genetically defined AML, frequently associated with altered differentiation	Mutant IDH produces 2-hydroxyglutarate, affecting α-ketoglutarate-dependent enzymes and epigenetic regulation; mutant-IDH inhibition can promote differentiation	[45,46]
PML::RARA-positive acute promyelocytic leukemia (APL)	Promyelocyte-stage differentiation arrest	Metabolic rewiring involving reduced glycolytic activity and increased reliance on fatty-acid oxidation, TCA-cycle activity and OXPHOS; altered arginine-associated metabolism	[41,47]
MLL-AF9-driven AML under stromal support or chemotherapy	Leukemic blasts exposed to microenvironmental and treatment-related cues	Stromal interactions can enhance TCA-cycle intermediates, antioxidant defenses and mitochondrial fitness	[48]
Cytarabine-resistant AML	Therapy-adapted cells; not necessarily functionally enriched for LSCs	Increased mitochondrial mass, membrane potential, oxidative metabolism and fatty-acid oxidation	[40]

Abbreviations: AML, acute myeloid leukemia; APL, acute promyelocytic leukemia; HSC, hematopoietic stem cell; LSC, leukemic stem cell; OXPHOS, oxidative phosphorylation; ROS, reactive oxygen species; TCA, tricarboxylic acid cycle.

## Data Availability

All data supporting the findings of this manuscript are contained within the manuscript.

## Abbreviations

The following abbreviations are used in this manuscript:

AA	Amino acid(s)
ACC	Acetyl-CoA carboxylase
ACSL1	Acyl-CoA synthetase long-chain family member 1
APL	Acute promyelocytic leukemia
AQP9	Aquaglyceroporin 9
AKT	Protein kinase B
ALL	Acute lymphoblastic leukemia
AMPK	AMP-activated protein kinase
AMPK1	AMP-activated protein kinase 1
AML	Acute myeloid leukemia
AMP	Adenosine monophosphate
ASS1	Argininosuccinate synthetase 1
ASL	Argininosuccinate lyase
ASNS	Asparagine synthetase
ATO	Arsenic trioxide
ATP	Adenosine triphosphate
ATRA	All-trans retinoic acid
BCAA	Branched-chain amino acid(s)
BCAT1	Branched-chain amino acid transaminase 1
BCL-2	B-cell lymphoma 2
BCL-XL	B-cell lymphoma-extra large
BH3	BCL-2 homology 3
BCT-100	Pegylated recombinant human arginase
BM	Bone marrow
BMI	Body mass index
CD34	Cluster of differentiation 34
CD36	Cluster of differentiation 36
CD38	Cluster of differentiation 38
CML	Chronic myeloid leukemia
CPT1	Carnitine palmitoyltransferase 1
DNA	Deoxyribonucleic acid
E3	E3 ubiquitin ligase
ETC	Electron transport chain
FA	Fatty acid
FADH2	Reduced flavin adenine dinucleotide
FAO	Fatty acid oxidation
FAS	Fatty acid synthesis
FASN	Fatty acid synthase
FH	Fumarate hydratase
FIS1	Fission, mitochondrial 1
FLT3	FMS-like tyrosine kinase 3
G6PD	Glucose-6-phosphate dehydrogenase
GLS1	Glutaminase 1
GLUT	Glucose transporter
GLUT1	Glucose transporter 1
GLUT3	Glucose transporter 3
GPx	Glutathione peroxidase
GSH	Glutathione
HAUSP	Herpesvirus-associated ubiquitin-specific protease
HDAC	Histone deacetylase
HIF-1α	Hypoxia-inducible factor 1 alpha
HK2	Hexokinase 2
HMG-CoA	3-Hydroxy-3-methylglutaryl-CoA
HSC	Hematopoietic stem cell
IDH	Isocitrate dehydrogenase
IDH1	Isocitrate dehydrogenase 1
IDH2	Isocitrate dehydrogenase 2
IDO1/2	Indoleamine 2,3-dioxygenase 1/2
ITD	Internal tandem duplication
LIC	Leukemia-initiating cell
LKB1	Liver kinase B1
LSC	Leukemic stem cell
MCL1	Myeloid cell leukemia 1
MCT1	Monocarboxylate transporter 1
MEIS1	Meis homeobox 1
Mgst1	Microsomal glutathione transferase 1
MLL-AF9	Mixed lineage leukemia–AF9 fusion
MYC	MYC proto-oncogene
NAD+	Oxidized nicotinamide adenine dinucleotide
NADH	Reduced nicotinamide adenine dinucleotide
NADPH	Reduced nicotinamide adenine dinucleotide phosphate
NK	Natural killer
NPM1	Nucleophosmin 1
NPM1c+	Cytoplasmic NPM1 mutant
NSG	NOD scid gamma
OCR	Oxygen consumption rate
OXPHOS	Oxidative phosphorylation
PDH	Pyruvate dehydrogenase
PDK	Pyruvate dehydrogenase kinase
PDK2	Pyruvate dehydrogenase kinase 2
PDK4	Pyruvate dehydrogenase kinase 4
PI3K	Phosphoinositide 3-kinase
PML	Promyelocytic leukemia protein
PPAR-δ	Peroxisome proliferator-activated receptor delta
PPP	Pentose phosphate pathway
PROTAC	Proteolysis-targeting chimera
PTEN	Phosphatase and tensin homolog
PUFA	Polyunsaturated fatty acid(s)
RARA	Retinoic acid receptor alpha
RNA	Ribonucleic acid
ROS	Reactive oxygen species
SAM	S-Adenosylmethionine
SCD	Stearoyl-CoA desaturase
SDHA	Succinate dehydrogenase subunit A
SLC1A5	Solute carrier family 1 member 5
SLC22A16	Solute carrier family 22 member 16
SOD	Superoxide dismutase
SRC-3	Steroid receptor coactivator 3
STAT3	Signal transducer and activator of transcription 3
STK11	Serine/threonine kinase 11
TCA	Tricarboxylic acid
TET	Ten-eleven translocation
TSC2	Tuberous sclerosis complex 2
Ub	Ubiquitin
USP7	Ubiquitin-specific protease 7
VDAC	Voltage-dependent anion channel
VLCAD	Very long-chain acyl-CoA dehydrogenase
